# First Steps Toward Ultrasound-Based Motion Compensation for Imaging and Therapy: Calibration with an Optical System and 4D PET Imaging

**DOI:** 10.3389/fonc.2015.00258

**Published:** 2015-11-30

**Authors:** Julia Schwaab, Christopher Kurz, Cristina Sarti, André Bongers, Frédéric Schoenahl, Christoph Bert, Jürgen Debus, Katia Parodi, Jürgen Walter Jenne

**Affiliations:** ^1^Mediri GmbH, Heidelberg, Germany; ^2^Department of Radiation Oncology, Heidelberg Ion-Beam Therapy Center (HIT), Heidelberg University Hospital, Heidelberg, Germany; ^3^Siemens Healthcare AG, Zurich, Switzerland; ^4^GSI Helmholtzzentrum für Schwerionenforschung, Darmstadt, Germany; ^5^Strahlenklinik, Erlangen University Hospital, Erlangen, Germany; ^6^Department of Experimental Physics – Medical Physics, Ludwig-Maximilian-University, Munich, Germany; ^7^Fraunhofer MEVIS, Bremen, Germany

**Keywords:** ultrasound imaging, ultrasound-based motion compensation, 4D PET imaging, ultrasound calibration, ultrasound in PET/CT

## Abstract

Target motion, particularly in the abdomen, due to respiration or patient movement is still a challenge in many diagnostic and therapeutic processes. Hence, methods to detect and compensate this motion are required. Diagnostic ultrasound (US) represents a non-invasive and dose-free alternative to fluoroscopy, providing more information about internal target motion than respiration belt or optical tracking. The goal of this project is to develop an US-based motion tracking for real-time motion correction in radiation therapy and diagnostic imaging, notably in 4D positron emission tomography (PET). In this work, a workflow is established to enable the transformation of US tracking data to the coordinates of the treatment delivery or imaging system – even if the US probe is moving due to respiration. It is shown that the US tracking signal is equally adequate for 4D PET image reconstruction as the clinically used respiration belt and provides additional opportunities in this concern. Furthermore, it is demonstrated that the US probe being within the PET field of view generally has no relevant influence on the image quality. The accuracy and precision of all the steps in the calibration workflow for US tracking-based 4D PET imaging are found to be in an acceptable range for clinical implementation. Eventually, we show *in vitro* that an US-based motion tracking in absolute room coordinates with a moving US transducer is feasible.

## Introduction

Permanent target motion, particularly in the abdomen, due to respiration or patient movement is still a challenge in many diagnostic and therapeutic procedures (1) and demands methods to detect and compensate this motion.

Especially in external beam radiation therapy, but also in diagnostic imaging, several approaches to avoid distorted images or substantial dose errors were proposed: mechanical motion mitigation via active breath hold or gating relative to the respiratory cycles are common ideas, which, however, extend treatment time and rely on the physical condition of the patient, as well as on a precise monitoring of the patient movement (2, 3). Several groups investigated motion detection by breathing belts or optical systems (4–6). These methods can detect irregularities like coughing or heavy breath takes but they only describe external motion and cannot observe the actual positions of inner organs. An example used in clinical practice is the breathing belt of the Respiratory Gating System AZ-733V (ANZAI Medical Co., Ltd., Tokyo, Japan), which only yields 1D tracking information of the outer abdominal movement.

Especially for tumor therapy with actively scanned ion beams, adaptive motion tracking (7, 8) promises to be fast and accurate at the same time, but this requires elaborate patient models that combine the external motion information to internal organ motion. Fluoroscopy offers the possibility to visualize inner structures, but it should not be used continuously during treatment because of the radiation burden (9). The *Calypso System* (Calypso Medical Technology, Seattle, WA, USA) used in prostate RT utilizes implanted RF-transponders for continuous motion tracking of the tumor (10). However, in this case, small beacons have to be implanted accurately near the tumor as fiducials.

An absolute, non-invasive, real-time capable method to monitor inner organs and register organ motion without any exposure to ionizing radiation would be the use of diagnostic ultrasound (US) imaging (sonography). It could be used continuously to detect the motion of a tumor either directly or by observing surrogate surrounding organs, for example, vessels (11) or the diaphragm (12). First experiments have shown that diagnostic US can be implemented successfully to radiosurgery using the CyberKnife (Accuray Inc., Sunnyvale, CA, USA) (13, 14). However, these two approaches rely on the tracking of fiducial markers, which might need to be implanted to the patient. The *Clarity* system (Elekta AB, Stockholm, Sweden), e.g., uses sonography to support inter-fractional positioning and recently to detect intra-fractional displacements of the prostate with a fixed US probe and at a rather low frame rate (2 Hz). In contrast to this quasi-static approach, our goal is to develop an US-based motion tracking method for real-time motion correction in 4D positron emission tomography (PET) imaging as used, e.g., for radiation therapy planning and verification. In addition, we want to consider a moving US tracking probe, e.g., attached to the patient skin and moved due to respiration, which requires a previous calibration of the tracking system – as also performed by Bruder et al. (15) for the CyberKnife.

The aim of this work was to integrate US-based motion monitoring to 4D PET imaging. This work is split into four parts: first, optical and US tracking systems were calibrated in order to provide absolute coordinate information independently of a moving US probe. Second, the US tracking was applied to 4D PET imaging and compared to the commercial ANZAI system, which is used in clinical practice. Third, artifact effects of the US probe in the PET/computed tomography (CT) field of view were investigated. Finally, US tracking in absolute coordinates was performed during 4D PET imaging in order to test the feasibility of the proposed workflow and the reliability of this new experimental setup.

## Methods

### Calibration of US Tracking System

**Figure 1:**
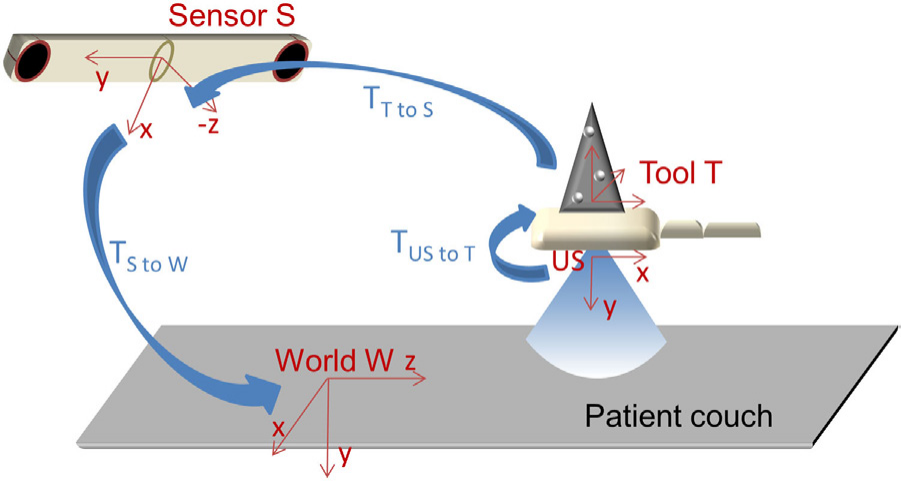
**The four involved coordinate systems in ultrasound motion tracking:** ultrasound image plane US (*z*_US_ = 0), optical marker tool T, optical sensor S as well as treatment room (“world”) W, and the transformations between them.

The optical US motion tracking setup comprises an US tracking system with a probe that is coupled to an optical marker as well as an optical tracking system, which detects the probe motion (see Figure 1).

When organ motion is detected by an optical US tracking system, there are four coordinate systems involved (see Figure 1): the one of the ultrasound images, the one of the optical marker tool T that is mounted statically on the US probe, the one of the optical sensor S, and the world coordinate system W, e.g., the treatment room. A point *p*_US_ is transformed to world coordinates as follows:

(1) $$p_{\text{W}}=T_{\text{S to W}}\cdot T_{\text{T to S}}\cdot T_{\text{US to T}}\cdot p_{\text{US}}$$

A coordinate transformation from system A to system B is described by the affine 4 × 4 transformation matrix *T*_A to B_. In order to perform a real-time coordinate transformation from US to W, we designed and implemented an all-in-one software application, which was used for both the calibration procedures as well as for the tracking.

**Figure 2:**
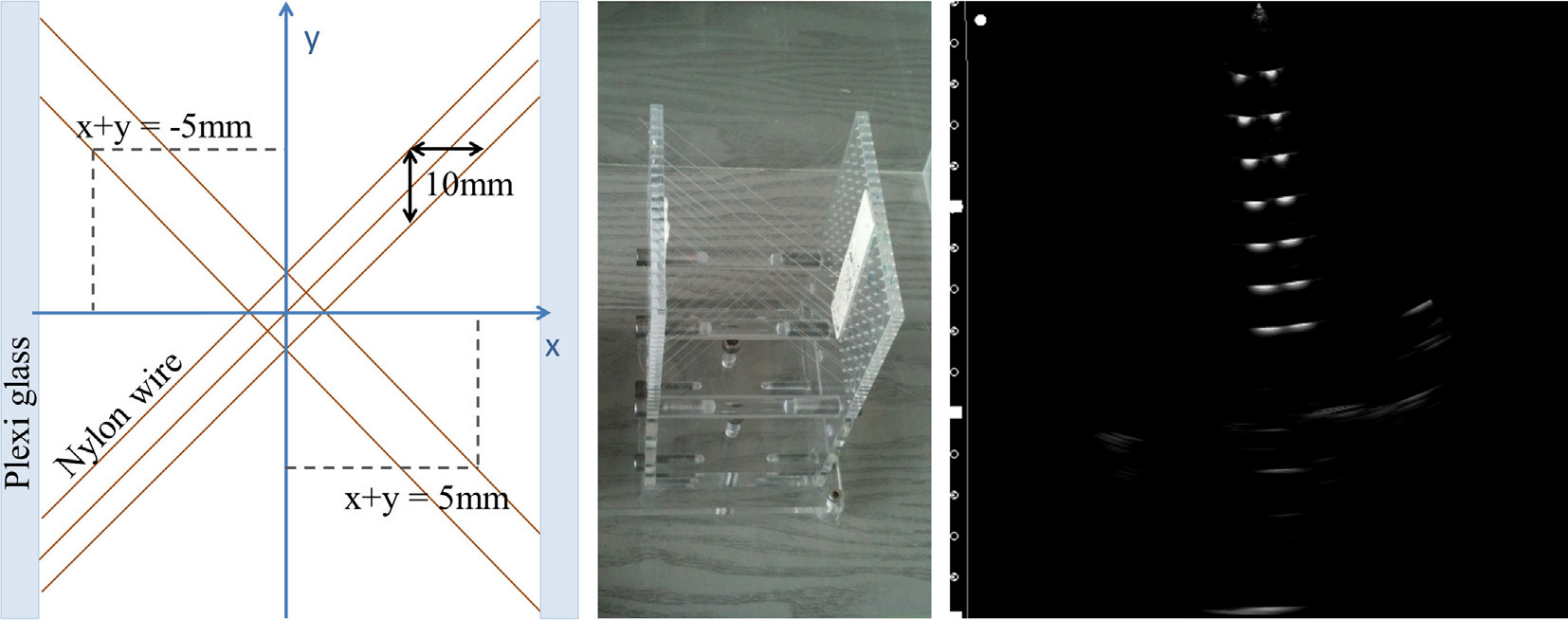
**The multi-cross wire water phantom**. Schematic view from the top (left) and photograph (middle) as well as a typical ultrasound scan of the wire construction imaged from the top (right).

For free-hand US calibration, a precisely manufactured phantom in a water bath (see Figure 2) is imaged several times with the US probe that is simultaneously tracked by an optical measurement system. To make sure that the acquired measurements yield a distinct (bijective) solution, all six degrees of freedom in the probe motion had to be taken into account and, thus, it was necessary to include multiple US images taken from different probe positions and orientations in the calibration process. The size of the water bath and the construction of the phantom allowed a broad polar and azimuthal imaging angle of the US probe.

The points *p*_US_ on the US images, representing the phantom structure, are correlated to their position *p*_W_ in the world coordinate system as described in Eq. (1). Using the known transformation *T*_T to S_, which reports the corresponding position and orientation of the probe, the transformations *T*_US to T_ and *T*_S to W_ can be determined by optimization calculation. The ratio of distances in real world to pixel in the US image (mm/pix) is included in *T*_US to T_. Due to the US image sector angle of 60°, it is the same in the *x*- and *y*-direction of the image.

The US images and the corresponding position of the US probe were recorded and evaluated automatically. In total, 10 equivalent calibration measurements (to check the reproducibility), each consisting of 64 US images were performed. In previous experiments, the number of 64 images was found to be a good compromise between precision and feasibility of the calibration. The results were checked for their reconstruction accuracy and calibration reproducibility (precision) similarly to the methods described in (16). It is our understanding that the accuracy of a measurement describes the degree of closeness of the measurement result to its true value. Whereas the precision stands for the degree to which repeated measurements under unchanged conditions show the same results. The accuracy of the calibration result for *T*_US to T_ was determined in additional measurements using a point phantom in the water bath. To compute the precision, random points were projected from US to T using all 10 calibration results, respectively. Then, the variance in their positions was determined.

In this work, the world coordinate system was identical to the treatment room coordinates of a commercial PET/CT scanner (PET combined with CT). The transformation T_S to W_ between the 3D coordinates of the optical sensor and the world coordinate system was determined by placing an optical marker tool at defined positions on the patient table and recording the corresponding position of the tool (at rest) in the optical sensor system. Thus, every position of the tool could be determined in both the two relevant coordinate systems and yielded three equations for the optimization of *T*_S to W_.

The patient table of the PET/CT could be moved automatically with a precision of 0.1 mm in two directions (up and down, in and out of the bore). Motions in the third direction (*x*_W_-axis of the PET/CT) were performed by hand with the aid of a ruler, which was mounted statically on the patient table. The precision of this supported free hand movement was 0.5 mm. To calibrate the optical sensor, in total, seven different runs, each with 10 different points in space, were conducted. To calculate the point reconstruction accuracy, each one of the seven measurement data sets served as cross-validation test data for the remaining six optimized transformation matrices. The calibration reproducibility (precision) was calculated by transforming three arbitrary but fixed points from the sensor coordinate system to the world coordinates using the seven optimized transformation matrices, respectively. The averaged deviations of the transformed points from their mean were used as estimation for the precision.

### Integration of US Tracking to 4D PET Image Reconstruction

**Figure 3:**
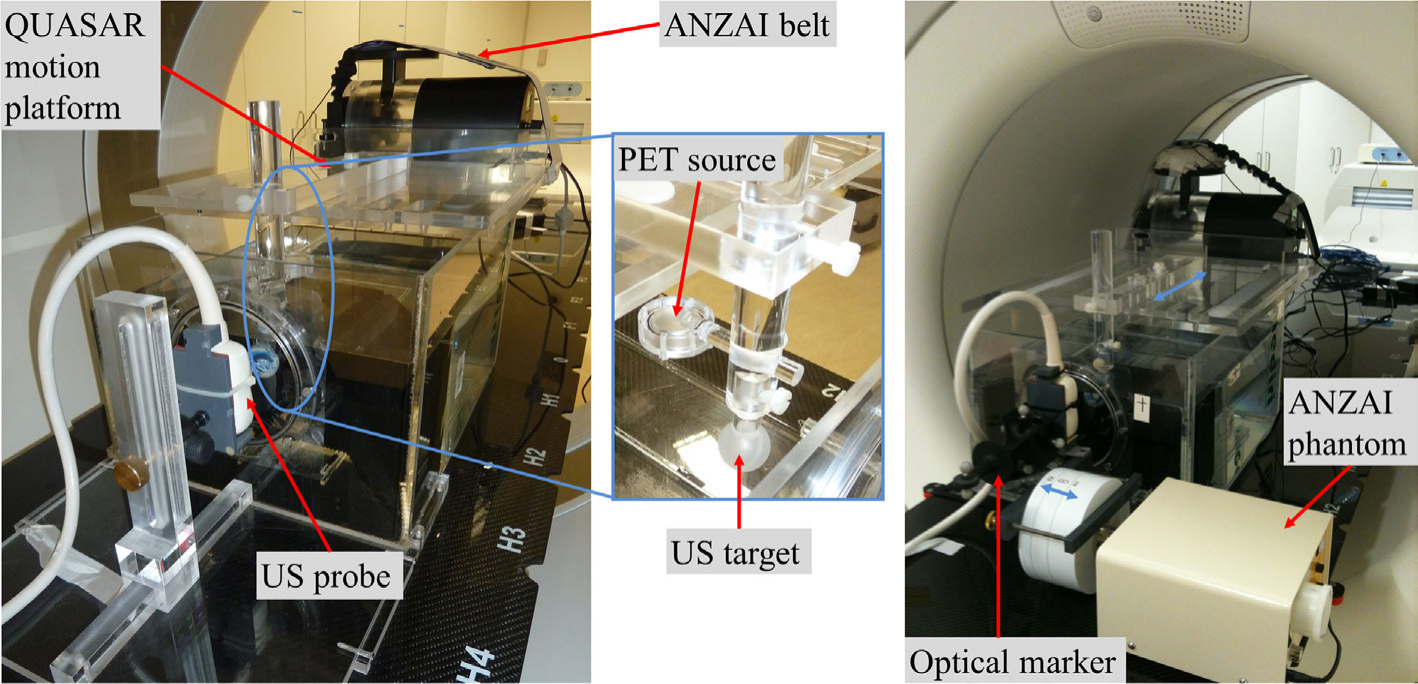
**Experimental setup for ultrasound-based motion tracking with static US probe (left) and with the US probe being moved by the ANZAI respiratory phantom (right) (from the optical sensor’s point of view)**. The setup was positioned on the patient table of the PET/CT scanner. The US target (rubber ball) and the rigidly attached PET ^22^Na point source were moved by the QUASAR motion platform. Motion was detected by the ANZAI breathing belt and the US probe in parallel.

In this part of the study, motion compensation in 4D PET imaging based on the presented US tracking system was compared to the performance of a commercial breathing belt. The experimental setup is shown in Figure 3 (*left*). A point source was moved along the PET/CT scanner axis by a respiratory motion phantom. A rubber ball was rigidly attached to the point source and put into a water-filled tank, which the US probe was coupled to through a Mylar foil window. Motion was simultaneously detected by the breathing belt, directly at the motion phantom as a standard reference, and by the US system, tracking the contour of the rubber ball. The whole setup was placed in the bore of the PET/CT scanner. A regular cosine^4^-shaped motion pattern with a peak-to-peak amplitude of 3 cm and a period of 4 s as well as a real patient motion trajectory with a maximum peak-to-peak amplitude of 3 cm were investigated. The latter one was recorded once during a real 30-min 4D patient PET/CT scan using the breathing belt and could be reproduced by the motion phantom. All trajectories were one dimensional along the scanner axis and inside the bore.

The US tracking algorithm *(12*)** uses active contours *(17*)** and conditional density propagation *(18*)**. Active contours, also called snakes, are deformable splines, which are often applied to noisy 2D images for delineation of object outlines. Conditional density propagation (“Condensation”) is then employed to track this contour. Based on the brightness values of an initially segmented target contour on the current US image, the algorithm yields five coordinates describing the position (translation in *x*-/*y*-direction), orientation (rotation within *x–y* plane) and scaling (scaling in *x*-/*y*-direction) of the target structure in real-time.

Positron emission tomography data were acquired every single millisecond in list-mode (LM) format with time tags. To enable a 4D image reconstruction, the positions in time of the inhale peaks, usually provided by the ANZAI respiratory gating system, were written into the acquired LM data stream as the so-called gate-tags. In the performed gated 4D PET image reconstruction (which is presently the only available time-resolved reconstruction opportunity on the scanner used), the LM data are subdivided into a user-defined number of phases between each two gate-tags on the basis of phase sorting: this means that the data between two inhale peaks are split into equal time bins. Each phase is reconstructed separately, resulting in a significant reduction of the point-source motion in each phase, but also in a decrease of the number of counts and herewith a decrease of the signal-to-noise ratio. In this study, 4D PET LM data have been subdivided into eight phases, as typically done in 4D patient examinations, and reconstructed by a filtered back-projection. Image reconstruction included attenuation correction based on a CT scan that was acquired prior to the measurement. The CT scan was a so-called free-breathing CT taking into account the complete experimental setup that was in the bore. The separately reconstructed PET images of the eight motion phases have then been manually registered to a common reference phase, chosen as the first phase after the maximum inhale peak, summed up, and divided by the number of phases. In contrast to the standard ANZAI motion surrogate, the US tracking device cannot be coupled directly to the PET/CT scanner. Instead, the US motion signal was acquired in parallel on a separate computer system and merged into the acquired LM data retrospectively. For this, the inhale peaks in the US tracking signal, considering only the displacement parameter along the scanner (*z*_W_-) axis, have been determined, corrected for the time offset between the two computer systems and were used to replace the ANZAI gate-tags within the acquired LM data. The temporal offset was computed by averaging the temporal shifts between corresponding inhale peaks of both motion monitoring systems. The inhale peaks were determined by means of Gaussian fitting. The manipulated LM stream, now containing US-based gate-tags, was then fed back in the PET/CT scanner, and reconstructed in the same way as the original LM data with ANZAI gate-tags. This enables a direct comparison of 4D-gated PET based once on the new US tracking device and once on the reference ANZAI system. The quantity of interest in this comparison was chosen to be the width of the point source in the direction of motion (along the scanner *z*-axis) in the reconstructed image, determined by a Gaussian fit at the *x/y* position of maximum activity.

### Investigations of Artifact Effects of the US Probe in the PET/CT Field of View

**Figure 4:**
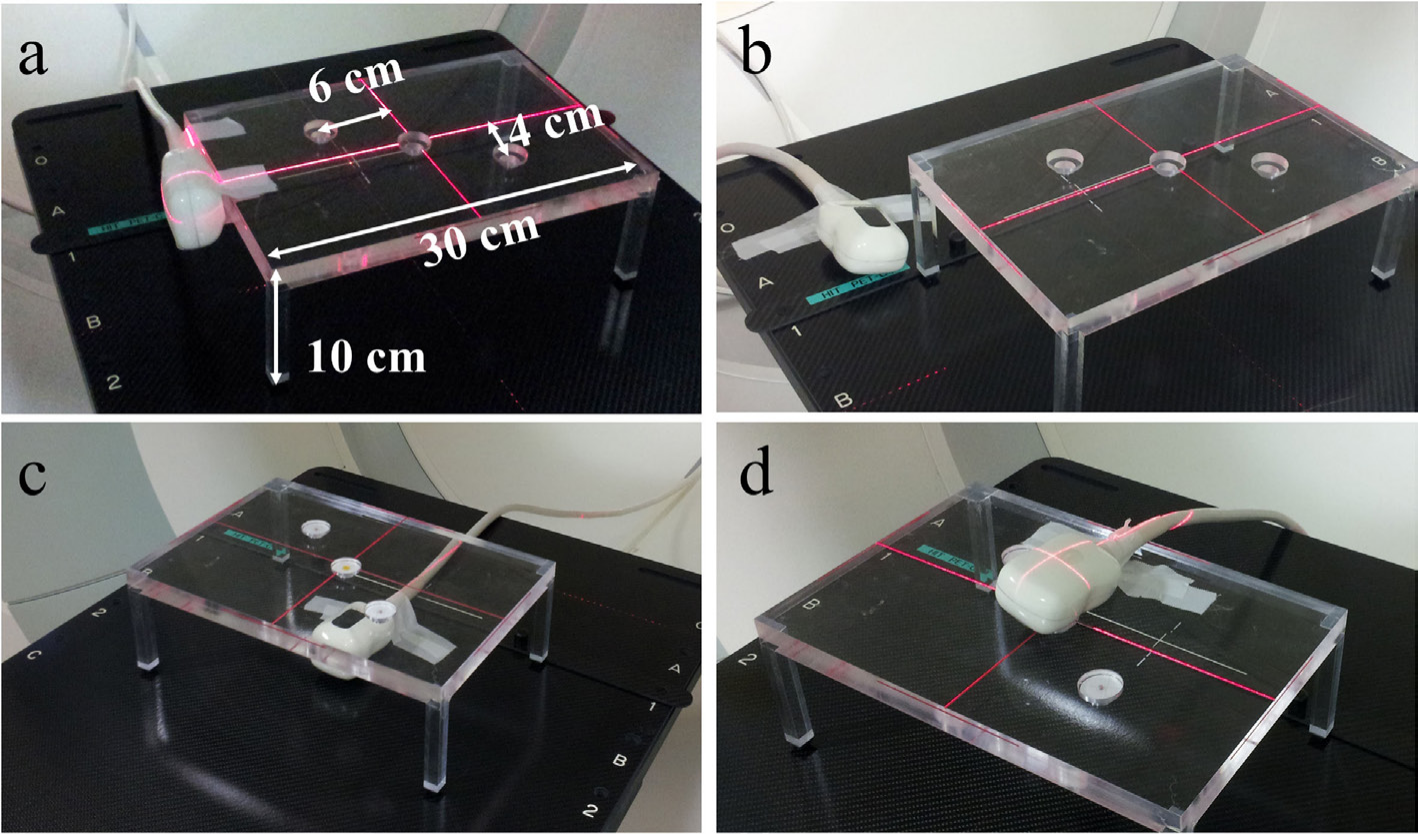
**Experimental setup for investigation of the US transducer’s influence on PET image reconstruction**. The three radioactive point sources were aligned in a horizontal diagonal line within the acrylic glass table. The measurement was repeated with the transducer in four different positions: **(A)** next to table (edge), **(B)** next to table (corner), **(C)** under table, and **(D)** on table.

In order to investigate the artifact effects of the US probe being within the lines of response of the PET detector ring, PET images of three radioactive point sources were acquired while the US probe was positioned close by the sources within the PET field of view. As shown in Figure 4, the three point sources were positioned in a horizontal diagonal line within a fixed small acrylic glass table construction, which was aligned with the laser cross hairs of the PET scanner. Five measurements were performed: one reference measurement without the US transducer and four measurements with the US transducer being fixated at different positions relative to the point sources. The four US probe positions were (a) next to the acrylic glass table in a central position at its edge, (b) next to the acrylic glass table close to a corner, (c) under the acrylic glass table, thus, approximately 6 cm under the central point source, and (d) lying on the table directly over the central point source. For each setup, an attenuation correction CT was acquired so that the US probe was taken into account during PET image reconstruction. The measured activities as well as the relative positions of the three point sources in the reconstructed image were compared, respectively.

### Ultrasound-Based Motion Tracking with a Moving Probe *In Vitro*

The last part of this work was performed to test the feasibility of the whole calibration procedure as well as the US tracking in absolute coordinates using the optical tracking system. Therefore, a similar setup as described in section 2.2 was used. However, as shown in Figure 3 (*right*), the US probe in front of the water tank was mounted on an additional motion phantom. It moved the probe sinusoidally (*A* = 20 mm peak-to-peak, *T* = 4 s) along the (horizontal) *x*_W_- axis of the treatment room coordinate system and, thus, orthogonally to the target motion. The setup was positioned on the patient table. The optical sensor was mounted statically on a tripod in front of the PET/CT scanner. The PET and US data were acquired simultaneously during 12 min, which means that 240 periods of the target motion (*A* = 40 mm peak-to-peak along *z*_W_, *T* = 3 s) were included.

As the radioactive point source was coupled rigidly to the moving US target, the transformed US tracking data could be compared to the position data of the reconstructed 4D PET images considering two constant offsets: the shift between rubber ball and radioactive point source was determined from the CT image of the setup and the distance between the PET coordinates and the world coordinates was defined by the manufacturer.

## Experimental Setup

The US tracking system used in this study is called Sonoplan II. It is developed by mediri GmbH, Heidelberg, Germany, and based on DiPhAS (digital phased array system, Fraunhofer IBMT, St. Ingbert, Germany). The US probe includes two 5.5 MHz phased array transducers (each with 64 elements), which are aligned perpendicular to each other (in one probe), allowing simultaneous imaging of two image planes. For optical tracking of the US probe motion, the Passive Polaris Spectra measurement system (Northern Digital Inc., Waterloo, ON, Canada) was used.

The calibration of the US tracking system was performed using a precisely manufactured water phantom. As shown in Figure 2, this multi-cross wire phantom consists of 29 nylon wires clamped between two acrylic glass plates with a distance of 80 mm. Figure 2 (*right*) shows a typical US image of the wire phantom. Every bright point in the US image representing a phantom wire yielded two equations, which could be fed into the Levenberg–Marquardt optimization algorithm (19, 20) implemented in our software.

The combined PET/CT scanner that was used during this study is a Biograph mCT, manufactured by Siemens Molecular Imaging, Knoxville, TN, USA.

Two respiratory motion phantoms have been used during this study. To move the radioactive point source (PET marker) and the rubber ball (US marker), the commercial QUASAR respiratory motion platform (Modus Medical Devices Inc., London, ON, Canada) was employed. For the last part of this study, the US probe itself was moved by the motion phantom of the Respiratory Gating System AZ-733V (ANZAI Medical Co., Ltd., Tokyo, Japan). The breathing belt was also part of the ANZAI Respiratory Gating System.

The radioactive point sources employed in this study were ^22^NA point sources or different activities.

## Results

### Calibration of the Ultrasound Tracking System

The precision of the US calibration was 1.0 ± 0.5 mm and the accuracy was 4.7 ± 2.0 mm. The calibration of the optical tracking system in the treatment room yielded an accuracy of 0.8 ± 0.2 mm and a precision of 0.5 ± 0.3 mm. The calibration results showed to be independent of the specifically chosen 10 measurement positions, as long as these were spread widely over the accessibly measurement volume. Thus, the overall accuracy of the tracking system was 4.8 ± 2 mm and its precision was 1.1 ± 0.6 mm.

### Integration of Ultrasound Tracking to 4D PET Image Reconstruction

**Figure 5:**
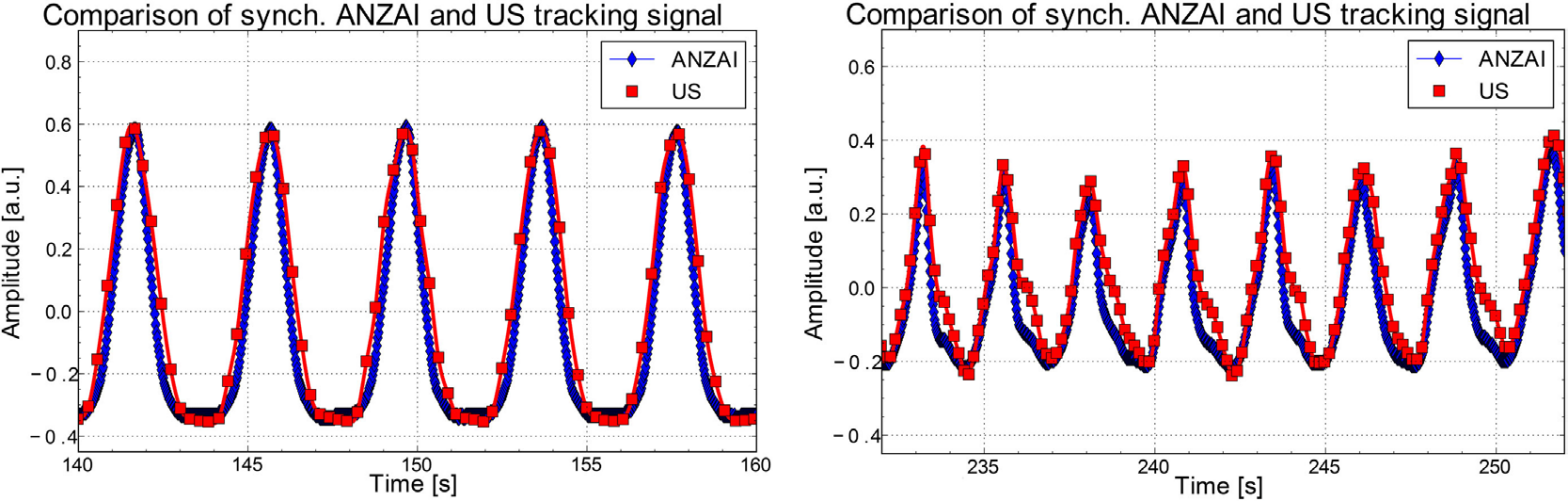
**Comparison of US- (squares) and ANZAI-detected (rhombi) motion trajectory**. As the US system provides a considerably lower frame rate, the data have been interpolated. A generally good agreement between the two data sets was found. Particularly the positions in time of the inhale peaks agree precisely, typically within 100 ms.

The acquired tracking data of the US system and the ANZAI surrogate are compared to each other in Figure 5. Of the 10 available tracking parameters determined by the US device for both imaging planes, only the displacement in the direction of motion (*z*-axis of the PET scanner), used in the retrospective LM data manipulation, is depicted. The other parameters were found to be constant in time for the selected one-dimensional motion aligned to the perpendicular US planes. As shown for both investigated motion patterns, a good agreement between the two tracking systems was found. Minor deviations were typically seen in the exhale part of the trajectory. In the performed 4D-gated PET image reconstruction, however, only the positions of the inhale peaks were of importance. Here, a typical deviation in time of less than 100 ms was found for all the investigated breathing patterns.

**Figure 6:**
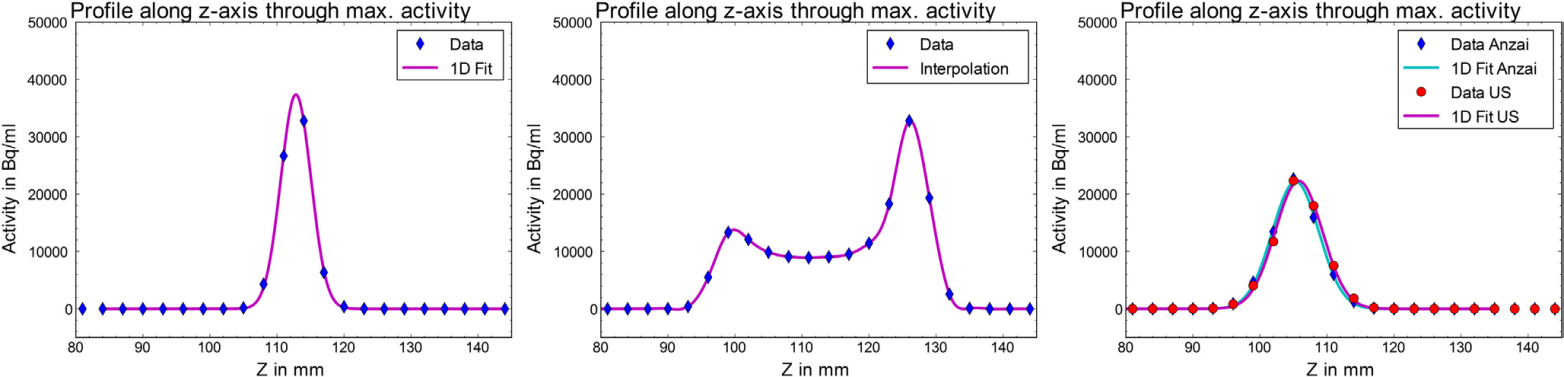
**Activity profiles of static (*left*) and moving (cosine^4^) point source in PET images**. Middle: the activity of the source is smeared over the whole motion amplitude (here 3 cm) if not corrected for. Right: using the 4D gated reconstruction based on the breathing belt (blue rhombi) as well as the ultrasound (red dots) signal, the Gaussian shape and integral activity can be recovered.

As shown in Figure 6 for the cosine^4^ motion, movement of the point source led to a considerable smearing of the point-like activity in the direction of motion and to a remarkably larger integral activity in the 3D reconstructed image due to the reduced partial volume effect. If, on the other hand, a 4D-gated image reconstruction was performed, motion-induced blurring was significantly reduced and the original Gaussian shape of the point source as well as the correct integral activity was recovered. This is shown in Figure 6 (*right*) for both considered motion monitoring systems, the breathing belt and the US tracking. Still, compared to the static reference, the full width at half maximum (FWHM) increased from 5.2 ± 0.2 (1σ) to 8.2 ± 0.2 (1σ) mm due to the residual motion within each of the eight considered motion phases.

An overview of the FWHMs obtained by the 1D Gaussian fit along the direction of motion in the 4D-reconstructed PET images, based once on the ANZAI and once on the US tracking signal, is presented in Table 1, together with the SDs of the above-mentioned time differences between ANZAI and US inhale peaks. The depicted FWHM values have an uncertainty of 0.2 mm originating from the manual registration of the reconstructed phases to the chosen reference phase, in addition to the uncertainty of the performed fit. An error of 0.2 mm in the manual registration process was estimated by multiple registrations of the same data set and comparison of the determined FWHMs. The error in the 1D Gaussian fitting was typically below 0.1 mm. Taking these uncertainties into account, a very good qualitative agreement between the clinically used ANZAI gating system and the US tracking system was found. As expected, a higher motion amplitude generally resulted in a larger FWHM due to an enhanced residual motion in the single breathing phases. Concerning the patient-like data set, it has to be considered that the average breathing amplitude was about 2 cm, i.e., smaller than maximum peak-to-peak amplitude of 3 cm. The breathing period, on the other hand, did not affect the results because of the used phase-based sorting of the LM data.

**Table 1:** **Overview of the determined point-source FWHMs and the standard deviations of the inhale peak time differences**.

Motion shape	Sinusoidal	Cosine^4^	Cosine^4^	Cosine^4^	Patient-like
	
	*T* = 4 s	*T* = 4 s	*T* = 4 s	*T* = 2.5 s	*A*_max_ = 3 cm
	
*A* = 4 cm	*A* = 3 cm	*A* = 2 cm	*A* = 3 cm
FWHM_Z_ (millimeter)	9.6	8.2	7.2	8.3	7.3
ANZAI FWHM_Z_ (millimeter)	9.6	8.3	7.4	8.3	7.5
US STD (Δ*t*) (millisecond)	50.0	32.3	41.4	33.6	81.1

*The combined error of the manual registration and the Gaussian fit in the FWHMs was determined to be 0.2 mm. Within this error, the results retrieved with breathing belt and US tracking agree for all investigated cases*.

### Investigations of Artifact Effects of the Ultrasound Probe in the PET/CT Field of View

The applicability of US tracking for 4D PET reconstruction under the aspect of the US probe being in the detector field of view was tested. The measured activities of the three point sources were compared for the diverse transducer positions (Table 2). The values for each point source varied only slightly, up to 3.5%. There is one exception case for the central point source when the US probe was put directly on top of it. Here, an overcorrection in the reconstruction causes a deviation relative to the reference activity of 20.7%. Furthermore, the geometric distortions of the reconstruction were analyzed. Therefore, the top left point source was chosen as fixed point and always registered to its reference position. The deviations of the other two point sources from their reference position served as quantification of the image distortion. As can be seen in Table 3, the maximum deviation was 2.2 mm, which, however, is still below the PET voxel size.

**Table 2:** **Measured activities of the three point sources (top left, middle, bottom right) for the four different US transducer positions compared to the reference without transducer**.

	Top left (10^8^ Bq)	Middle (10^7^ Bq)	Bottom right (10^8^ Bq)
Reference	2.95	8.25	2.93
Next to table (edge)	3.01 (+2.0%)	8.17 (−1.0%)	2.83 (−3.4%)
Next to table (corner)	2.89 (−2.0%)	8.23 (−0.2%)	2.91 (−0.7%)
Under table	2.99 (+1.4%)	8.23 (−0.2%)	2.88 (−1.7%)
Over table	2.86 (−3.1%)	9.96 (+20.7%)	2.93 (+0.0%)

**Table 3:** **Geometric distortion caused by the US transducer being within the lines of response of the PET scanner**.

	Middle (**Δ***x*, **Δ***y*, **Δ***z*) (millimeter)	Bottom right (**Δ***x*, **Δ***y*, **Δ***z*) (millimeter)
Next to table (edge)	0.0, 0.8, 0.3	−0.2, 2.2, 0.5
Next to table (corner)	0.3, 0.0, 0.3	0.6, 0.0, 0.8
Under table	0.4, 0.0, 0.4	0.6, 0.0, 0.8
Over table	0.1, 0.0, 0.3	0.6, 0.0, 0.8

*The top left source was always registered to its reference position. The numbers represent the deviations of both the other sources from their reference position for all four transducer positions*.

### Ultrasound-Based Motion Tracking with a Moving Probe *In Vitro*

**Figure 7:**
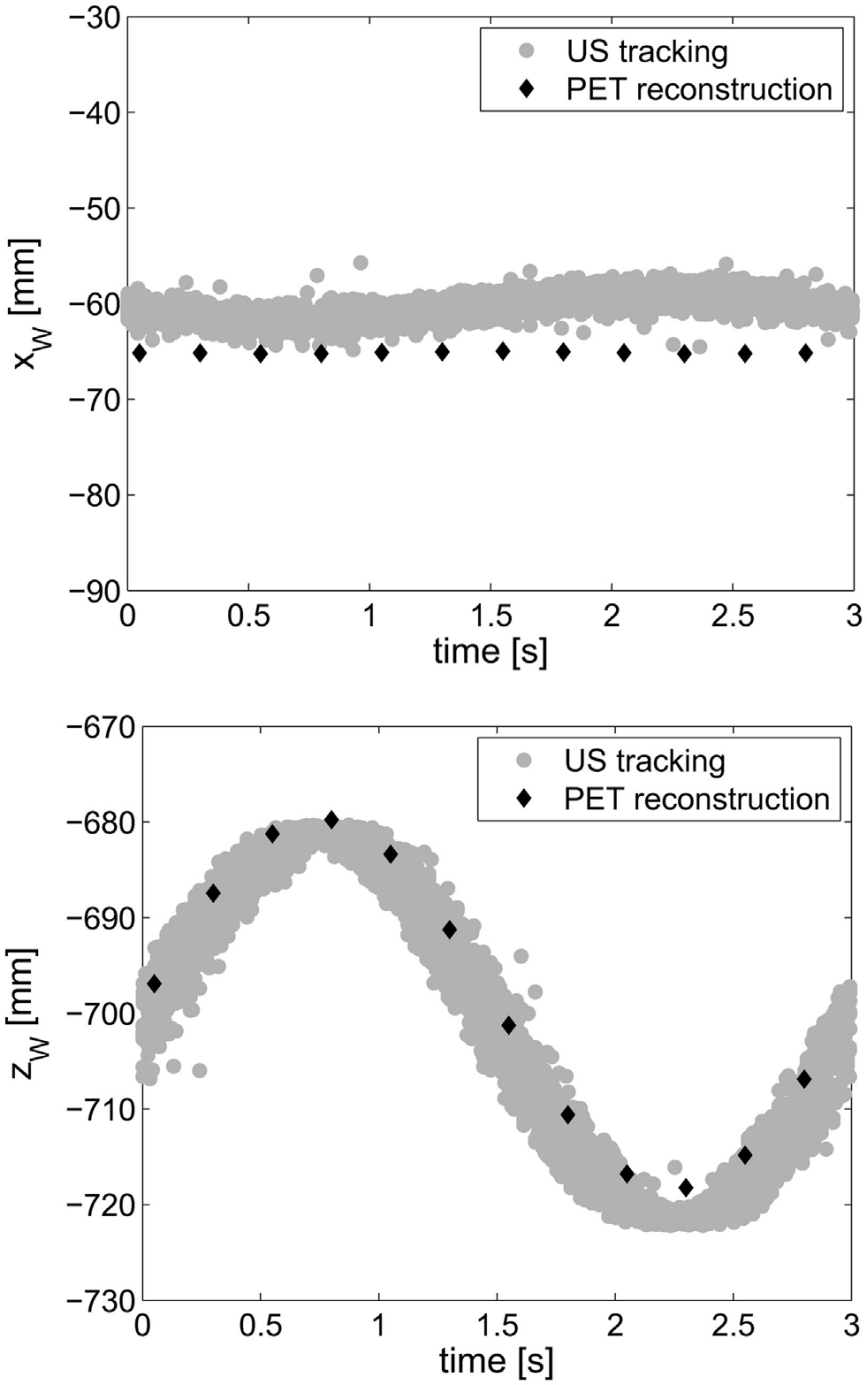
**Ultrasound tracking data transformed to world coordinates (gray dots) and PET reconstruction data along the *x*_W_- and *z*_W_-axes**. The PET data have been corrected for the constant offsets both between rubber ball and radioactive point source as well as between the PET coordinates and the world coordinates.

The setup could be installed at the PET scanner without problems, and both, the optical and the US tracking systems, performed as expected. The overall frame rate for the transformed tracking data was approximately 10 Hz due to the frame rate of the optical tracking, which we did not succeed to raise during this study. In Figure 7, the transformed US tracking data are plotted together with the PET reconstruction data along the *x*_W_- and *z*_W_-axes of the treatment room (world) coordinate. The US tracking data (gray dots) has a variance of 0.7 and 2.2 mm in *x*_W_- and *z*_W_-direction, respectively. These values range within the accuracy of the US calibration, which was found to be below 4.8 mm. The average positions of the point source for each of the 12 considered phases are plotted as black rhombi.

As shown in Figure 7 (top), there is a discrepancy in the PET and US data of 4.8 ± 1.0 mm along the *x*-axis of the treatment room coordinates. This ranges within the accuracy of the US tracking system. Although the target was not moving along the *x*_W_-axis, the US tracking data show a residual motion in the *x*_W_-direction of ±2 mm with 3-s period. However, this is 80% less than the actual motion of the probe in *x*_W_-direction of ±10 mm. In Figure 7 (bottom), the measured target motion has the expected amplitude of 40 mm. The US and PET data coincide very well.

## Discussion

### Calibration of the Ultrasound Tracking System

The calibration of the US system was performed with a multi-cross wire water phantom inspired by other multi wire and point phantoms (21–25). It combines the simplicity of a single-point phantom with the possibility of semi-automated segmentation.

The US calibration was performed at a penetration depth of 140 mm, which would be reasonable for abdominal applications. The accuracy was 4.8 mm and 4.9 mm for image planes 1 and 2, respectively. Considering the probe architecture with 64 elements in each of the two arrays and the large penetration depth, which yields a relatively poor resolution in the images, this is a reasonable value. The precision of the presented calibration method was determined to 1.0 and 1.5 mm for image planes 1 and 2, respectively. Hsu et al. (26) used another multi-wire phantom to perform a free-hand US calibration and achieved an accuracy of 3.0 mm and a precision of 1.2 mm for a curvilinear probe with a penetration depth of 150 mm. In the literature, various values that seem to describe a better performance with higher accuracy and precision can be found (16, 27). However, in most cases, they are obtained at a penetration depth of only about 30 mm and in addition to a higher frequency, which may be a reason for a higher resolution and better results.

For future works, a new phantom that can be scanned from even more diverse positions and orientations could help enhance the quality of the calibration. An alternative approach would be to integrate the optical marker in the construction plan of the US probe and manufacture it with adequate precision. Employing another transducer with a higher number of elements or using higher harmonics could also enhance the US image accuracy and, thus, the accuracy of the whole calibration.

The optical tracking system was calibrated such that it could yield the position and orientation of any optical marker tool within the measurement volume of the sensor not only in the sensor coordinate system but also in target space, i.e., treatment room coordinates. The accuracy of the presented calibration method is 0.8 ± 0.2 mm and the precision is 0.5 ± 0.3 mm. This is slightly below the volumetric accuracy that is reported by the manufacturer of the optical sensor. They determined the measuring accuracy to 0.30 mm (28). The accuracy and precision of the overall calibration procedure were determined to be 4.8 ± 2 mm and 1.1 ± 0.6 mm, respectively. This is fully acceptable for PET image reconstruction considering that it refers to absolute treatment room coordinates and yields *in situ* tracking information. In light of radiotherapy, it might be necessary to further improve the accuracy based on the amendments mentioned above.

### Integration of Ultrasound Tracking to 4D PET Image Reconstruction

In a first experimental study with moving ^22^Na point sources, a good agreement of the motion trajectories, simultaneously detected by the standard ANZAI pressure surrogate and the prototype US tracking system, was found. The method of retrospectively replacing the ANZAI gate-tags in the acquired LM PET data by the determined US gate-tags proved to work reliably. Concerning the motion mitigation in time-resolved PET imaging of moving point sources, an equivalent performance of both systems could be demonstrated (Figure 6). If no motion correction was applied, the activity distribution in the reconstructed 4D PET image showed the expected smearing and a higher integral activity, which was due to the reduced partial volume effect of the moving point source. If, however, motion correction was applied, the original Gaussian shape of the activity distribution could be recovered. As the first phase after the inhale peak was chosen as reference phase, the position of the activity distribution was slightly shifted toward “exhale” in the motion-corrected cases (Figure 6*right*).

A previous synchronization of each independent data set was found to be necessary in order to correct for the observed, non-constant time offset between the two different operating systems, which the tracking routines were run on. Consequently, the time offsets had to be determined at the beginning of each single PET acquisition. This problem can likely be solved by running the US tracking directly on the ANZAI computer, which was not feasible in this study as the ANZAI computer is in clinical use and additional software installation not allowed. As reported in (29), part of the found time offsets might also be attributed to delays in the US tracking software. It was, however, shown that these delays can be overcome by an artificial neural network motion prediction.

In the presented results, a superiority of the US tracking system could not be shown due to the chosen setup and the used gated 4D PET image reconstruction, only relying on the position of the gate-tags, i.e., the inhale peaks, and not on the actual source position. In order to demonstrate the promised advantages of US tracking, a more detailed investigation with a more complex, 3D point-source motion, and a more sophisticated way of sorting the acquired LM data into the different motion phases, making use of all 10 provided US tracking parameters, would be of need and will be tackled in forthcoming studies.

### Investigations of Artifact Effects of the Ultrasound Probe in the PET/CT Field of View

The influence of the US transducer being within the detector ring of the PET scanner showed to play a minor role in the image reconstruction. The induced changes in the measured activity of three point sources were all below 3.5%, which is only marginal. There was only one exception when the probe was lying directly on the central source. Here, the activity was overestimated in the reconstruction by 20.7%. However, this was caused by an overcorrection of the actually measured activities due to artifact effects of the US probe in the attenuation correction CT. These artifact effects will decrease when the CT is acquired with additional tissue (e.g., a patient) in the scanner. Furthermore, the geometric distortion in the reconstructed images due to the US probe was found to be smaller than 2.2 mm, which is negligible compared to the voxel size of the PET scanner.

### Ultrasound-Based Motion Tracking with a Moving Probe *In Vitro*

In this experiment, the validity of the calibration and the practicality of the overall workflow were assessed. The setup showed that the proposed method allows real-time tracking in absolute coordinates even if the US probe was moved, e.g., by using an adhesive probe attached to the patient’s skin.

The presented data prove that the main motion of the target is reproduced in the correct direction with the expected amplitude regardless of the probe moving or standing still. The probe motion could be mitigated by 80% (from 20 to 4 mm) due to the optical tracking. Taking into account that the chosen motion amplitudes represented the maximum values observed in respiratory motion, this result is quite promising. The frame rate should indeed be enhanced, however, 10 Hz are already sufficient for respiratory motion, which is in the scale of some seconds. Although the overall accuracy of the tracking system is only slightly below 5 mm, it is still acceptable taking into account that the tracking information is 2D and is acquired *in situ*. Clinically established non-ionizing portable systems mostly track surrogates or the patient surface and yield 1D information. For phase-based gated PET imaging as it is performed at the moment, this information might be sufficient. However, as soon as 4D-PET/CT systems are able to exploit tracking information from the complete amplitude, potentially in two dimensions, a comprehensive US-based tracking system will be beneficial. Abdominal structures as the liver vessels or the diaphragm allow for good 2D tracking not only of breathing motion but also of extraordinary patient movement, which then could be accounted for by advanced reconstruction algorithms of the imaging modality.

In the future, the presented optical US tracking system may be integrated into any time-resolved imaging process, such as 4D treatment planning or *in vivo* PET validation (30). Also, the presented method may find application in gated radiotherapy (31) or in conjunction with actively scanned ion-beams (29). To further exploit the opportunities and advantages of US tracking, it would be interesting to use both image planes of the T-probe or even a 3D transducer but this is beyond the scope of this report. Moreover, a registration process that fits a previously determined 3D model of the target to the actual US slice would enhance the procedure. Furthermore, an MRI compatible version of DiPhAS is being developed by mediri GmbH and Fraunhofer IBMT (32). The system is shielded with copper and development versions of the US probe can be attached to the patient’s skin as a sticker.

## Conclusion

In this work, a combined optical US tracking system for motion compensation in diagnostic and therapeutic systems with a moving probe was calibrated, implemented to 4D PET imaging, and evaluated. The accuracy and precision of all necessary calibration steps were found to be promising for clinical use. The functionality of all hardware and software components was tested in a proof of principle *in vitro* experiment to examine the overall reliability and feasibility of the proposed calibration workflow. Our initial study showed that 4D PET imaging based on US motion tracking is feasible. In a first experimental campaign, we could show that results equivalent to the clinically used ANZAI respiratory gating system could be achieved in 4D-gated PET. Further studies with more complex motion patterns, particularly with uncorrelated motions in more than one dimension, should aim to show the anticipated benefits from US motion tracking.

## Conflict of Interest Statement

The authors declare that the research was conducted in the absence of any commercial or financial relationships that could be construed as a potential conflict of interest.
